# Multidimensional tracking of phenotypes and organ involvement in a complete nationwide systemic sclerosis cohort

**DOI:** 10.1093/rheumatology/keaa026

**Published:** 2020-02-25

**Authors:** Håvard Fretheim, Anne-Kristine Halse, Marit Seip, Helle Bitter, Marianne Wallenius, Torhild Garen, Anne Salberg, Cathrine Brunborg, Øyvind Midtvedt, Øyvind Molberg, Anna-Maria Hoffmann-Vold

**Affiliations:** k1 Department of Rheumatology, Oslo University Hospital – Rikshospitalet, Oslo, Norway; k2 Institute of Clinical Medicine, University of Oslo, Oslo, Norway; k3 Department of Rheumatology, Haukeland University Hospital, Bergen, Norway; k4 Department of Clinical Science, University of Bergen, Bergen, Norway; k5 Department of Rheumatology, University Hospital of North Norway, Tromso, Norway; k6 Department of Rheumatology, Hospital of Southern Norway, Kristiansand, Norway; k7 Department of Rheumatology, St. Olav’s University Hospital, Trondheim, Norway; k8 Department of Neuromedicine and Movement Science, Norwegian University of Science and Technology, Trondheim, Norway; k9 Department of Rheumatology, Lillehammer Hospital for Rheumatic Diseases, Lillehammer, Norway; k10 Oslo Centre for Biostatistics and Epidemiology, Research Support Services, Oslo University Hospital, Oslo, Norway

**Keywords:** systemic sclerosis, epidemiology, pulmonary hypertension, interstitial lung disease, gastrointestinal involvement

## Abstract

**Objective:**

SSc is a severe, heterogeneous multi-organ disease where population-based estimates on phenotypic spectrum, overall disease burden and societal impact are largely missing. Here the objective was to provide the first-ever complete national-level data on phenotype and major organ afflictions in SSc.

**Methods:**

A stepwise strategy was applied to find and characterize every SSc patient resident in Norway from 2000 to 2012. First we identified every case in the country registered with an International Classification of Diseases, Tenth Revision code for SSc (M34). Next we manually reviewed all cases coded as M34 to determine whether they met the 1980 ACR and/or 2013 ACR/EULAR classification criteria for SSc and could be included in the Norwegian SSc cohort (Nor-SSc). Finally, all disease features from SSc onset to study end were reviewed.

**Results:**

The Nor-SSc cohort included 815 SSc patients. The mean age at diagnosis was 53 years, with 84% females and 77% limited cutaneous SSc. The estimated incidence increased from 4 per million in 2000 to 13 per million in 2012. We identified high cumulative frequencies of internal organ involvement, coexistence of multiple organ afflictions across disease subsets and autoantibody status and stable frequencies of pulmonary arterial hypertension across haemodynamic definitions, but indications of referral-related differences in pulmonary hypertension detection rates across the study area.

**Conclusion:**

This nationwide cohort study provides new, unbiased evidence for a high disease burden in SSc patients of Caucasian descent and indicates the existence of hurdles preventing equality of assessment across the SSc population.

**Rheumatology key messages**

- Incidence and prevalence rates of systemic sclerosis increased over the study period of 13 years.
- There was a high cumulative coexistence of multi-organ disease in SSc patients.
- The percentage of SSc patients referred to right heart catheterization varied across the study area.

## Introduction

SSc is a heterogeneous disease with large interindividual differences in skin involvement, internal organ afflictions, disease progression and outcome [1–7]. An increasing number of randomized clinical trials (RCTs) in SSc has been conducted in recent years and despite their limitations, they are the gold standard research method for determining clinical management [8–12]. However, other approaches, including long-term follow-up population-based studies, would be more suitable in many SSc related research issues. Obvious examples are critical outcome measures like mortality, target organ damage, risk of cancer and cardiovascular disease, health-related quality of life and societal burden of disease. The same holds true when studying the discrepancy of conducted diagnostic testing and treatment practices within a defined study area. Moreover, there are still substantial knowledge gaps in SSc epidemiology and how the spectrum of the disease is distributed in unselected cohorts.

Inherent properties of the health care system makes Norway one of the few countries worldwide were it is possible to establish nationwide population based cohorts [13, 14]. In Norway, there is universal public health care and all patient contacts are registered in electronic patient journal systems using the International Classification of Diseases, Tenth Revision (ICD-10) coding system (the ICD system has been in use since 1999). Since every inhabitant in Norway has a unique 11-digit personal identification (ID) number, there is no loss to follow-up during observation. Rheumatology departments at public hospitals, of which there are 17, including 4 at university hospitals, have the primary responsibility for SSc care in Norway, and our previous work from the south-east Norway area showed that every SSc patient in that area was followed at a department of rheumatology [14]. Finally, Norway is a relatively small country, which in January 2013 had 5.05 million inhabitants (86% of Norwegian ethnicity, 6% Western and 8% non-Western immigrants). These circumstances make it fully feasible to identify every single diagnosed patient resident in Norway within a defined time period.

In the present study, we aimed to include all SSc patients in Norway in a prospective, nationwide Norwegian SSc (Nor-SSc) cohort to provide national-level unbiased epidemiologic data on SSc, estimate the frequency of organ afflictions, with special emphasis on pulmonary hypertension, and establish a platform for understanding the overall impact of SSc on the society.

## Methods

### Stepwise case-finding strategy

The method was partly described previously [15]. In detail, we captured all SSc patients resident in Norway between 2000 and 2012 and applied a stepwise strategy. In 2013 we searched through the administrative databases of all public hospitals in Norway and the databases of the 12 private rheumatologists in the country to identify all patient contacts coded by ICD-10 as M34.0, M34.1, M34.8 and M34.9 (SSc) at least once during the study period. Next we used the 11-digit Norwegian personal ID number system to control for duplicate registrations and patients with registrations at more than one location. Additionally we identified cases that were already included in the prospective Oslo University Hospital SSc cohort and registered in the Norwegian Systemic Connective Tissue Disease and Vasculitis Registry (NOSVAR) registry.

In the second step, performed from 2013 to 2017, we reviewed in detail the electronic patient journal of every patient who was resident in Norway between 2000 and 2012 and registered at least once with an M34 code, recording relevant disease parameters in a predefined patient form. In patients who were already included in the NOSVAR, we added registry data to the electronic patient journal data and merged these in the patient form. The major purposes of this detailed review were to determine whether the patient met the study cohort inclusion criteria (see below) and to retrieve and systematically record longitudinal data on disease features from the time of diagnosis to the end of the inclusion period on 1 January 2013, or death.

### Inclusion and exclusion criteria for the Nor-SSc cohort

Patients identified by the stepwise case-finding strategy were included in the Nor-SSc cohort if they met the following criteria: age >18 years, clinical SSc diagnosis, fulfilment of the 1980 ACR and/or 2013 ACR/EULAR SSc classification criteria and findings not better explained by another disease. We included patients with overlap diseases if they met these criteria, but excluded all M34-coded patients who had connective tissue diseases other than SSc, localized scleroderma or morphea.

This study complied with the Declaration of Helsinki. The Regional Committee of Health and Medical Research Ethics in south-east Norway approved the study and received exemption of informed consent for identification of the patients and chart review (2009/1035).

### Recording of demographic data and disease features ever present

For each patient, we recorded available data on the time of onset of RP and the first non-RP symptom, time of clinical SSc diagnosis, SSc subset defined as sine scleroderma SSc, lcSSc and dcSSc [16]. Data on modified Rodnan skin score and nailfold capillaroscopy were registered [17–19]. Digital ulcers, calcinosis, telangiectasia, scleroderma renal crisis (SRC), tendon friction rub, dysphagia, diarrhoea, obstipation and faecal incontinence were recorded in the patient chart if ever present. Myositis was evaluated by clinical diagnosis and/or positive biopsy; gastroesophageal reflux disease was evaluated by upper endoscopy and/or pH measurement and/or patient-reported symptoms of gastroesophageal reflux disease. Oesophagus dysmotility was defined by dynamic X-ray and gastric antral vascular ectasia (GAVE) by upper endoscopy [9, 20, 21].

Data from serum autoantibody tests were obtained by a review of each patient’s laboratory results. Methods for the detection of ANA were IIF or ELISA. ACA and anti-topoisomerase antibodies (ATAs) were always detected by ELISA. Anti-RNA polymerase III, anti-fibrillarin, anti-Th/To, NOR90, U1-RNP, kU and PmScl75/100 were detected by immune blot (SSc blot, Euroimmun, Luebeck, Germany) and scored according to the manufacturer’s protocol.

### Assessment of cardiopulmonary involvement, including pulmonary hypertension

Echocardiography parameters, 6 min walking distance test (6MWD), N-terminal pro-brain natriuretic peptide (NT-proBNP), pulmonary function tests and high-resolution CT (HRCT) lung images from baseline and the last available follow-up visit were noted [14, 22]. All HRCTs were reviewed manually as described [22]. Reticular pattern abnormalities and superimposed ground-glass opacities were defined as equivalent to fibrosis and the extent of pulmonary fibrosis was expressed as a percentage of total lung volume [23]. Pulmonary function tests with diffusing lung capacity for carbon monoxide (DLCO), forced vital capacity (FVC) and forced expiratory volume during the first second (FEV1) were carried out according to American Thoracic Society/European Respiratory Society (ERS) guidelines as described [22]. Right heart catheterization (RHC) was registered if conducted and pulmonary hypertension (PH) was diagnosed according to the 2015 European Society of Cardiology/ERS guidelines as mean pulmonary arterial pressure (mPAP) ≥25 mmHg and borderline PH as mPAP 20–24 mmHg [24, 25]. Patients were further classified as pre- and post-capillary PH, based on a threshold pulmonary capillary wedge pressure (PCWP) of 15 mmHg [24, 26]. Additionally we applied the new haemodynamic definitions for PH proposed by the 6th World Symposium on Pulmonary Hypertension (WSPH) with pre-capillary PH defined by mPAP >20 mmHg, PCWP ≤15 mmHg and pulmonary vascular resistance (PVR) ≥3.0 Wood units (WU) [27]. We also included analyses of lower PVR threshold values (PVR ≥2.5 and ≥2.0 WU). PAH [World Health Organization (WHO) group 1] was diagnosed as described [22, 28] by the presence of pre-capillary PH; the absence of significant interstitial lung disease (ILD), defined as <10% lung fibrosis by HRCT at baseline and follow-up investigations and/or by a predicted FVC >70% at baseline and follow-up; or exclusion of other pre-capillary PH causes. The PH-ILD (WHO group 3) diagnosis was defined as pre-capillary PH combined with lung fibrosis >10% on HRCT and/or FVC <70%. In the absence of RHC, possible PH was noted and defined as systolic pulmonary arterial pressure (sPAP) >40 mmHg on the echocardiography, annual decline in the DLCO >10% and/or unexplained functional class 4. Findings consistent with cardiovascular disease (angina pectoris and myocardial infarction) were recorded if ever present.

### Prevalence and incidence

Incident SSc cases were diagnosed from 2000 to 2012 while prevalent cases included patients diagnosed before 2000. The point prevalence of SSc was calculated from all incident and prevalent cases alive on 1 January 2013. We calculated the frequency of incident SSc patients year by year from 2000 to 2012. Incidence rates were expressed as the number of cases per million inhabitants >18 years of age per calendar year and presented with 95% CIs.

### Statistics

Registration forms were scanned (Cardiff TeleForm version 9) and manually checked and imported to an Access 2016 database (Microsoft, Redmond, WA, USA). Analyses were performed with SPSS version 25 (IBM, Armonk, NY, USA) and STATA version 15 (StataCorp, College Station, TX, USA). Descriptive data are presented as numbers and percentages, mean with s.d. or median with range. Comparisons between groups were evaluated with independent sample *t* test and Mann–Whitney *U* test, as appropriate. For analysing correlations, Pearson or Kendall’s τ_b_ coefficients were applied.

## Results

### Case-finding strategy, patient demographics and population prevalence of SSc

**Fig. 1:**
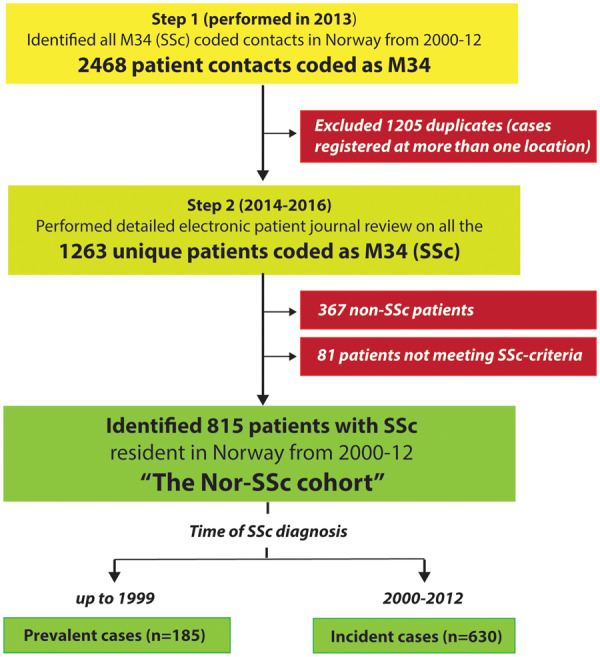
Case-finding strategy and patient inclusion in the Nor-SSc cohort Stepwise case-finding strategy (yellow boxes) and number of excluded patients (red boxes) and the number of identified SSc patients in the Nor-SSc cohort segregated by prevalent and incident cases (green boxes).

In the initial case-finding step, we searched across all patient administrative databases in the country and identified 2468 patient contacts registered at least once with an M34 code (M34.0, M34.1, M34.8 or M34.) in at least one database during the time period from January 2000 to December 2012. By merging all these M34-coded patient contacts against national ID numbers, we found that 1205 of the 2468 cases identified were registered in more than one database (i.e. at more than one location). Removal of these duplicate registrations left us with 1263 unique patients (Fig. 1).

In the second step, we performed a detailed electronic patient journal review on the 1263 unique patients who had been registered at least once with an M34 code between 2000 and 2012. A total of 367 of the 1263 patients had to be excluded because their clinical features were not compatible with SSc. Most of the excluded cases had an incident chronic rheumatic disease other than SSc and had been registered as M34 on a single occasion, typically early in their disease course. Additionally, we excluded 81 patients who had one or more SSc-associated disease feature but failed to meet the SSc classification criteria (demographics in online Supplementary Table S1, available at *Rheumatology* online). After having excluded these 448 (367 + 81) patients, we were left with a cohort of 815 cases who had clinical SSc by chart review and met all the Nor-SSc cohort inclusion criteria.

Of the 815 patients in the final Nor-SSc cohort, 630 (77%) were defined as incident cases (i.e. diagnosed from 2000 onwards) while 185 (23%) were prevalent cases (i.e. diagnosed before 2000). As expected from the structure of the Norwegian health care system, we found that the 815 patients in the cohort were primarily referred to a rheumatologist for diagnostic procedures and follow-up. It appeared that 404 of the 815 Nor-SSc patients (49.6%) were already included in the prospective SSc cohort at Oslo University Hospital [14].

**Fig. 2:**
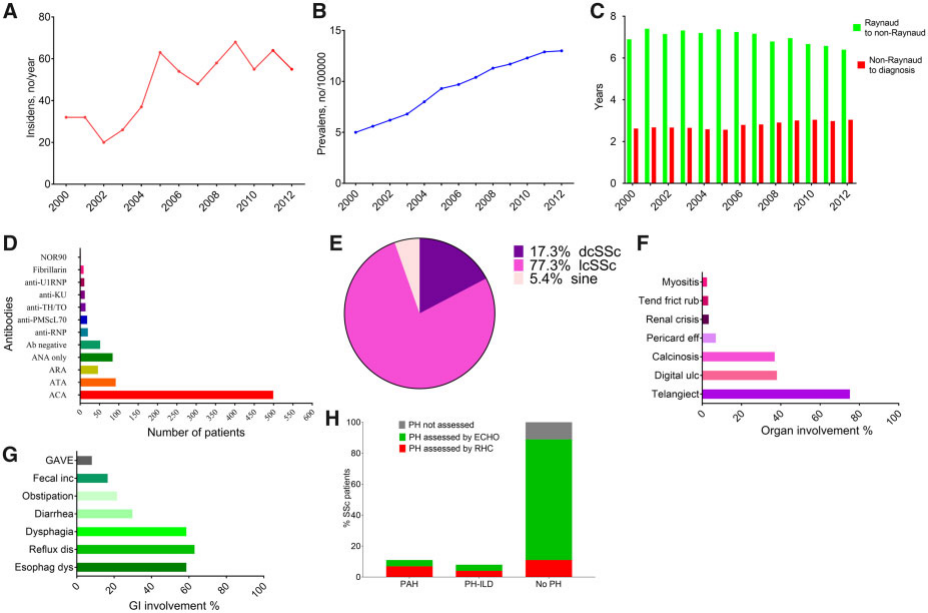
Prevalence and incidence in the Nor-SSc cohort (**A**) The number of newly diagnosed SSc patients per year from 2000 to 2012. (**B**) Point prevalence of SSc in Norway each year from 2000 to 2012. (**C**) Mean time (in months) from RP to first non-RP symptom (green) and time from first non-RP symptom to diagnosis (red). (**D**) Distribution of SSc-related serum antibodies in the cohort. (**E**) Distribution of SSc according to skin involvement. (**F**) Frequencies of skin and renal involvement and other organ manifestations. (**G**) Frequencies of GI involvement. (**H**) Assessment of pre-capillary PH, with estimates for definite PH diagnosed by RHC and possible PH by echocardiography and clinical parameters.

Patient demographics are shown in Table 1. By 1 January 2013 a total of 161 patients (19.9%) were deceased (Table 1). The estimated point prevalence of SSc in Norway on 1 January 2013 was 13/100 000 (95% CI 12.0, 13.9), with 5/100 000 (95% CI 4.1, 5.9) in males and 22/100 000 (95% CI 20.2, 23.9) in females. The number of newly diagnosed SSc patients per year increased from 20–42 cases each year in the time period 2000–2004 to 50–64 cases in 2005–2012 (Fig. 2A), corresponding to an estimated mean annual SSc incidence of 4–6 per million in the time period 2000–2004 and 10–13 per million in 2005–12.

**Table 1:** Demographics, key SSc features and physical function in the total Nor-SSc cohort segregated by SSc subtype

Characteristics	Total (*N* = 815)	dcSSc (*n* = 141)	lcSSc (*n* = 629)
Demographics			
Age at diagnosis, years, mean (s.d.)	53 (14.7)	49 (16.4)	54 (14.2)
Time from RP to non-RP, years, mean (s.d.)^a^	6.4 (10.3)	3.2 (9.0)	7.1 (3.4)
Time from non-RP to diagnosis, years, mean(s.d.)^b^	3.1 (5.1)	1.4 (3.0)	3.5 (5.5)
Female, *n* (%)	682 (83.7)	95 (67.4)	547 (87)
Observation period, years, mean (s.d.)	11.1 (7.7)	10.9 (9.2)	11.9 (7.4)
Deceased by 17 April 2017, *n* (%)	234 (28.7)	62 (44.0)	168 (26.7)
Key SSc features			
Modified Rodnan skin score, median (range)^c^	5.0 (0-49)	18.0 (1-49)	4 (0-18)
Abnormal nailfold capillaroscopy, *n* (%)^d^	498 (94.4)	81 (95.3)	399 (95)
Physical function			
Functional class 1 or 2, *n* (%)^e^	486 (59.6)	53 (28.5)	433 (68.8)
Functional class 3 or 4, *n* (%)^e^	112 (13.7)	23 (12.4)	89 (14.1)
6MWD, m, mean (s.d.)^f^	457 (149.6)	438 (161.4)	482 (145.9)
Analyses of pulmonary function tests			
FVC, % predicted, mean (s.d.)^g^	94 (20.9)	83 (22.6)	97 (19.7)
DLCO, % predicted, mean (s.d.)^g^	69 (20.2)	63 (20.1)	71 (19.8)

Data were complete except for the following:

a available in 573 patients,

b available in 731 patients,

c evaluated in 513 patients,

d evaluated in 525 patients,

e evaluated in 598 patients,

f evaluated in 379 patients and

g available in 703 patients.

### Overview of autoantibodies and cumulative incidence of disease features

Test results for ANAs were available in 99.6% of the cohort patients and were positive in 93%. The frequency of specific autoantibodies and cumulative incidences of specific organ manifestations are shown in Table 2 and Fig. 2D.

**Table 2:** Autoantibody profile and organ involvement in the total Nor-SSc cohort and segregated by SSc subtype

Characteristics	Available, *N* (%)	Total, *N* (%)	dcSSc (*n* = 141)	lcSSc (*n* = 627)
Antibodies	806 (98.9)			
ACA, *n* (%)		497 (61.7)	11 (7.8)	451 (72.9)
ATA, *n* (%)		91 (11.3)	47 (33.3)	45 (7.3)
Anti-RNAP3, *n* (%)		46 (5.7)	38 (27.1)	8 (1.3)
Organ involvement				
Lung fibrosis, *n* (%)	650 (79.8)	324(49.9)	94 (75.4)	217 (44.4)
Digital ulcers, *n* (%)	793 (97.3)	301 (38.0)	62 (44.6)	234 (38.4)
Calcinosis, *n* (%)	704 (86.4)	260 (36.9)	30 (24.4)	218 (40.7)
Telangiectasia, *n* (%)	739 (90.7)	556 (75.2)	79 (60.8)	449 (79.8)
SRC, *n* (%)	782 (96.0)	26 (3.3)	20 (14.5)	6 (1.0)
Tendon friction rub, *n* (%)	594 (72.9)	18 (3.0)	18 (18.2)	1 (0.2)
Myositis, *n* (%)^a^	753 (92.4)	18 (2.4)	8 (5.9)	9 (1.6)
Oesophagus dysmotility, *n* (%)^b^	664 (81.5)	531 (80.0)	109 (87.2)	404 (79.4)
GERD, *n* (%)^c^	683 (83.8)	430 (63.0)	80 (64.0)	335 (63.4)
Dysphagia, *n* (%)	732 (89.8)	429 (58.6)	78 (60.9)	328 (58.4)
Constipation, *n* (%)	608 (74.6)	131 (21.5)	32 (26.7)	95 (20.5)
Diarrhoea, *n* (%)	627 (76.9)	187 (29.8)	41 (34.2)	144 (29.8)
Faecal incontinence, *n* (%)	564 (69.2)	93 (16.5)	25 (22.9)	67 (15.4)
GAVE, *n* (%)^d^	456 (56.0)	37 (8.1)	13 (16.5)	24 (6.6)

a Evaluated by barium esophagram.

b Evaluated by clinical diagnosis and/or biopsy.

c Evaluated by finding on upper endoscopy and/or pH measurement and/or patient-reported symptoms of GERD.

d Diagnosed by upper endoscopy.

RNAP3: RNA polymerase III; GERD: gastroesophageal reflux disease, defined by patient-reported symptoms and/or findings on gastroscopy/pH measurement. % is calculated using number of patients with available data as the denominator.

Baseline lung CT data were available in 650 patients and lung fibrosis, of varying degree, was identified in 324 of 650 patients (50%). The mean baseline FVC was 94% (s.d. 20.9) and the mean baseline DLCO was 69% (s.d. 20.2), as previously published [15].

**Fig. 3:**
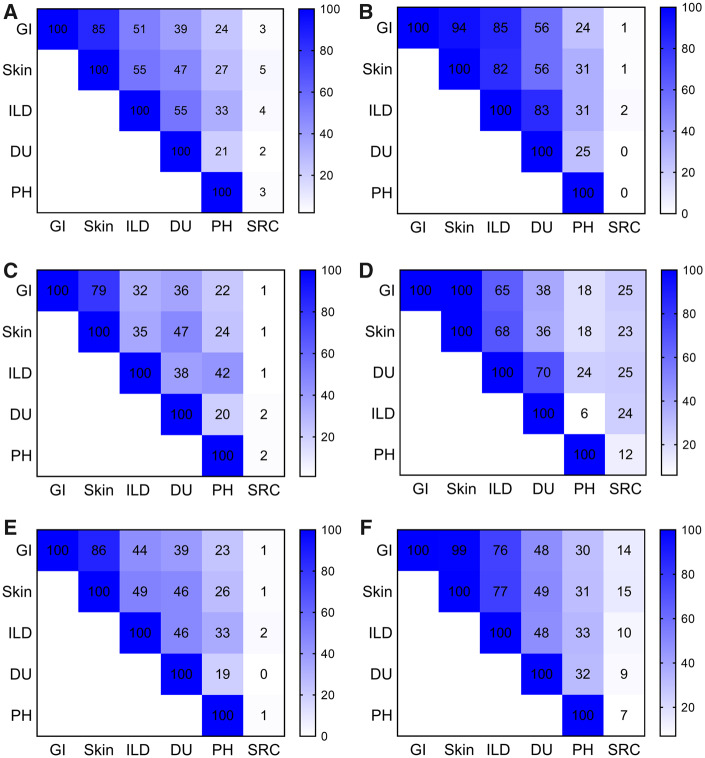
Cumulative coexistence of six major disease features Cumulative coexistence of six major disease features including GI involvement, skin affection, lung fibrosis, digital ulcers, PH and SRC stratified by antibody status or disease subtype with (**A**) all patients, (**B**) patients positive for anti-topoisomerase I antibody, (**C**) patients positive for ACAs, (**D**) patients positive for anti-RNA polymerase III antibody, (**E**) lcSSc patients and (**F**) dcSSc patients.

Multi-organ disease was highly common, with more than two of six features being major disease features [gastrointestinal (GI) involvement, skin affection, lung fibrosis, digital ulcers, PH and SRC] ever present in the vast majority of the cohort patients, also when stratified by antibody status or disease subtype (Fig. 3).

### Development of PH during the observation period

As the chart review process indicated differences in RHC referral practices by time and location (see below), we reasoned that it would be most appropriate to estimate accumulated PH frequencies at two levels: as definite PH verified by RHC and as possible PH indicated by sPAP >40 mmHg by echocardiography at baseline and/or follow-up and additional annual decline in DLCO >10% and/or unexplained functional class 4.

**Fig. 4:**
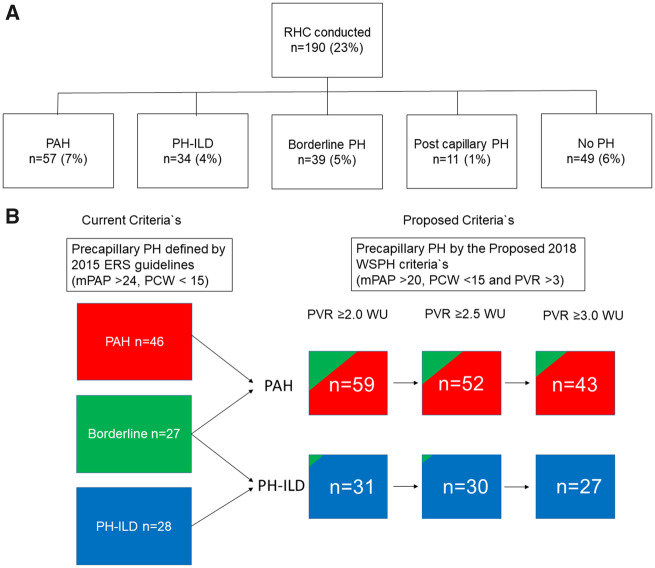
Frequency of PH assessed by RHC (**A**) Cumulative incidence of PH subsets during the study period according to the current PH definition. (**B**) Cumulative incidence of PAH, PH-ILD and borderline PH in patients with available PVR values (*n* = 155) and the number of patients fulfilling the newly proposed pre-capillary PH definition and PVR >2.5 WU and >2.0 WU.

PH assessment by RHC was conducted in 190 patients (23%) (Fig. 4), with 160 (84%) having the RHC procedure done at Oslo University Hospital. Thus the proportion of patients examined by RHC was higher in the Nor-SSc cohort subset ever examined at Oslo University Hospital [160/404 (40%)] than among the subset followed at any of the other hospitals [30/413 (7%); *P* < 0.001]. Additionally, it appeared that the annual number of RHC examinations increased through the observation period, from 1–10 RHCs per year in 2000–2008 to 10–15 per year in 2009–2012 and 15–22 in 2013–2016.

Pre-capillary PH by the 2015 guidelines was diagnosed in 91 of 190 patients subjected to RHC, with PAH in 57 (7%) and PH-ILD in 34 (4%). The mean time from disease onset to PAH diagnosis was 8.3 years (s.d. 8.4). A total of 155 patients had complete hemodynamic values (including PVR) available, allowing for pre-capillary PH evaluation by the new WSPH criteria (Fig. 4B). We found that the prevalence of PAH defined by mPAP >20 mmHg and PVR ≥3.0 WU was 5%. When lowering the PVR limit to ≥2.5 and ≥2.0 WU the prevalence increased to 6% and 7%, respectively. With PVR ≥3.0 WU as the cut-off, the frequency of PH-ILD cases was 3%, increasing to 4% when lowering the PVR value to ≥2.0 WU (Fig. 4B).

Baseline echocardiography was conducted in 728 patients (89%), with 103 (14%) having an estimated sPAP >40 mmHg. Analyses of available follow-up echocardiography in 398 patients (48%) showed that 61 patients (16%) with normal sPAP at baseline had developed new-onset sPAP >40 mmHg. Among the patients having an estimated sPAP >40 mmHg by echocardiography at baseline and/or follow-up, we identified 74 patients who had never been referred to RHC but had an annual decline in DLCO >10% and/or unexplained functional class 4 considered as compatible with PH. These 74 patients were defined as possible PH cases, with 44 classified as possible PAH and 30 as possible PH-ILD. By adding together the cases with definite pre-capillary PH by RHC (>25 mmHg) and possible PH, we found an estimated cumulative PH incidence in the Nor-SSc cohort of 19% (165/815), with 11% being PAH and 8% PH-ILD cases. This total estimate was identical to the pre-capillary PH frequency observed in the patient subsets with RHC from Oslo University Hospital (*n* = 160), but the relative frequency of PAH and PH-ILD in this subset was slightly different, with 12% having PAH and 7% PH-ILD.

Plasma NT-proBNP at baseline was available for 460 patients (56%) with a mean value of 86.2 pmol/l (s.d. 312.8). Follow up NT-proBNP was available for 367 patients (45%), with a mean value of 195.1 pmol/l (s.d. 684.2).

## Discussion

Studies of population-based patient cohorts derived from well-defined areas are needed to fully appreciate the overall impact of a disease. Here we present data from the first complete nationwide cohort study on SSc. Major findings were increasing incidence and prevalence rates of SSc throughout the study period, high cumulative coexistence of multi-organ disease and indications of referral-related differences in PH detection across the study area. Overall, we believe that the results provide unbiased evidence for a very high disease burden in SSc patients of Caucasian descent and indicate unmet needs for equality of assessment.

With entries for >16 000 SSc patients from >150 countries, the EULAR Scleroderma Trials and Research group (EUSTAR) database is definitely an immense source of knowledge and a unique platform for SSc research [7, 29–31]. Recruitment policies are heterogeneous across EUSTAR centres and there are no requirements to register all patients. A large proportion of centres, particularly the large centres, recruit all of their patients into the database, but it is not required. It is therefore unknown whether the spectre of disease seen in the EUSTAR patient population mirrors that of an unselected population-based SSc cohort [4, 32].

Although the estimated point prevalence of SSc at the study end was still within the range reported from regional studies performed in other northern European countries, we observed a steady increase in point prevalence of SSc throughout the 13 year study period [33]. We cannot exclude the possibility of a true increase in the incidence of SSc, but favour the explanation that it is due to increased SSc awareness throughout Europe in recent years. Thus it is noteworthy that the diagnostic delay (i.e. the time from the first SSc symptom to diagnosis) remained the same across the study period. This observation probably mirrors the referral delay recently shown by Distler *et al.* [34] and emphasizes the persisting educational needs of referring physicians and patients.

There is an ongoing discussion concerning the hemodynamic definitions for PH diagnosis, with recently proposed new definitions lowering the mPAP value to >20 mmHg and including a conservative PVR cut-off value ≥3.0 WU to capture all patients with manifest pre-capillary PH [27]. However, this threshold is arbitrary and it has been suggested that a PVR >2.0 WU should be regarded as abnormal [26]. Applying the proposed definitions with a PVR cut-off value of 3.0 WU did not have a major impact on the number of patients diagnosed with pre-capillary PH. Further studies are needed to explore whether a cut-off value of PVR ≥3.0 WU is appropriate in SSc patients.

Although the overall estimates of organ afflictions in Nor-SSc indicated that GI and cardiopulmonary involvement were highly common, we observed differences in certain afflictions, particularly PH, across the study area. More detailed analyses indicated that the cumulative incidence of PH varied between local and academic centres and was dependent on the number of RHCs conducted at the different centres. Screening recommendations for PH include annual echocardiography assessments, and RHC referral is indicated if there are abnormal echocardiography findings in the context of clinical PH suspicion [24, 26]. However, annual echocardiography and RHC is not easily available in all centres following SSc patients, and there are no existing data on how often echocardiography and RHC are conducted in academic compared with non-academic centres. These circumstances might have had implications for previously reported data regarding PH and indicate that we are still in need of increased PH awareness, better and standardized PH screening methods and guidelines on referral to expert centres for RHC independent of haemodynamic definitions of PH. The same argument is most likely valid for GI involvement, for which no generalized screening recommendations exist. This results in differences between centres, such as the varying frequency of GAVE screening by upper endoscopy noted across the Nor-SSc cohort. With the aim of standardizing the follow-up of patients with SSc, a Delphi-based expert consensus was recently published that will hopefully lead to an adequate standard of care for all patients with SSc and enhance the standardization and homogenization of the practices worldwide [35].

The main strengths of our study were the comprehensive inclusion and identification strategies. All diagnosed SSc patients in Norway were included in the Nor-SSc cohort and all diagnoses of every patient were confirmed by chart review. Due to the health system in Norway, we did not have any loss to follow-up and vital status was available for all patients. Additionally, a high percentage of patients had follow-up data available to estimate the longitudinal development of certain organ involvements. Despite the comprehensive and complex strategies applied to include all patients in the country, we still might have missed some patients with SSc due to miscoding. However, the fact that colleagues at Oslo University Hospital who applied similar ICD-10-based case-finding strategies for other connective tissues diseases (inflammatory myopathies, SLE and MCTD) did not find any SSc patients who were not already registered in the Nor-SSc cohort underscores the completeness of our cohort [36–38].

The main limitations were a study design that could only estimate the frequency of new SSc diagnoses per year, but not the true incidence. Additionally, we did not have complete coverage of longitudinal clinical data in all the patients, possibly influencing the prevalence of certain organ manifestations. Chart review was performed by a rheumatologist experienced with SSc, but clinical assessment in the different centres could have been performed by a rheumatologist with less experience. Finally, we might have missed patients with very mild forms of the disease, who do not necessarily seek health care but still could fulfil the 2013 classification criteria.

In conclusion, we provide the first real-life data on prevalence and incidence, organ involvement and the impact of SSc on a nationwide level. The results strongly support the notion that SSc is a rare, heterogeneous disease with a high disease burden. They also indicate that Norway, probably like most other countries, has not yet reached the aim of achieving equality of assessment and care throughout the nation.

## Supplementary Material

keaa026_Supplementary_Data
