# Supplementary material for: Multidimensional tracking of phenotypes and organ involvement in a complete nationwide systemic sclerosis cohort
Source: Rheumatology (Oxford). 2020 Feb 25;59(10):2920–9. doi: 10.1093/rheumatology/keaa026 (PMC7516103; doi:10.1093/rheumatology/keaa026)
Supplement: keaa026_Supplementary_Data [file keaa026_supplementary_data.docx]

**SUPPLEMENTARY MATERIAL**

**Supplementary Table S1 Demographics and key SSc features of the patients not fulfilling the SSc classification criteria**

| **Characteristics** | **Total**  **(n=81)** |
| --- | --- |
| Demographics |  |
| Age at time of first contact, years (SD) | 46 (14.1) |
| Female gender, n (%) | 71 (87.7) |
| Observation period, years (SD) | 5.1 (4.3) |
| Key SSc features |  |
| Raynauds phenomenon, n (%) | 77 (95.1) |
| Anti-nuclear antibody, n (%) | 79 (97.5) |
| Puffy hands, n (%) | 6 (7.4) |
| Abnormal nailfold capillaroscopy, n (%) | 71 (87.7) |
| SSc specific antibodies, n (%) | 60 (74.1) |

SSc: systemic sclerosis; SD: Standard deviation; n: number
